# Supplementary material for: Phytoene and Phytoene-Rich Microalgae Extracts Extend Lifespan in C. elegans and Protect against Amyloid-β Toxicity in an Alzheimer’s Disease Model
Source: Antioxidants (Basel). 2024 Jul 31;13(8):931. doi: 10.3390/antiox13080931 (PMC11351246; doi:10.3390/antiox13080931)
Supplement: Supplementary file 1 [file antioxidants-13-00931-s001.zip › antioxidants-3051544-supplementary.pdf]

Compound/ Extract	Temp.	FUDR (μM)	Deaths/ censored	Mean lifespan (days)	Lifespan change (%)	*p* vs control
Control	20˚C	100	**[1-4] 279/81**	**16.05**		
			[1] 53/7	16.36		
			[2] 75/15	17.24		
			[3] 77/13	16.15		
			[4] 74/46	14.48		
C. sorokiniana	20˚C	100	**[1-4] 111/130**	**17.66**	**+10.02**	**<0.0001**
			[1] 34/26	20.10	+22.82	0.0014
			[2] 56/34	17.97	+4.23	<0.0001
			[3] 10/21	17.25	+6.80	<0.0001
			[4] 11/49	16.73	+15.58	<0.0001
D. bardawil	20˚C	100	**[1-4] 110/189**	**19.18**	**+19.50**	**<0.0001**
			[1] 32/28	20.79	+27.05	0.0015
			[2] 23/36	19.52	+13.26	<0.0001
			[3] 30/60	18.31	+13.34	<0.0001
			[4] 25/65	19.07	+31.76	<0.0001
Phytoene	20˚C	100	**[1-4] 91/123**	**18.58**	**+15.79**	**<0.0001**
			[1] 28/35	21.30	+30.17	0.0026
			[2] 32/28	18.60	+7.88	<0.0001
			[3] 23/37	17.93	+10.98	<0.0001
			[4] 8/23	18.00	+24.34	<0.0001

Table S1. Lifespan data of C. elegans treated with *C. sorokiniana* and *D. bardawil* extracts and phytoene. 1-4 shows combined data from all trials.
